# Assessment of ventriculo-vascular properties in repaired coarctation using cardiac MRI derived aortic, left atrial and left ventricular strain

**DOI:** 10.1186/1532-429X-17-S1-P207

**Published:** 2015-02-03

**Authors:** Quanliang Shang, Shivani Patel, Phalla Ou, David Danford, Andreas Schuster, Philipp B Beerbaum, Samir Sarikouch, Shelby Kutty

**Affiliations:** 1 University of Nebraska Medical Center, Chiildren's Hospital and Medical Center, Omaha, NE USA; 2 University Paris Diderot, Hopital Bichat, APHP, Paris, France; 3 University Medical Center, Göttingen, Göttingen, Germany; 4 Hanover Medical University, Hannover, Hannover, Germany

## Background

Impaired arterial strain is associated with increased stiffness in the great arteries. In patients with repaired coarctation of the aorta (CoA), we sought to assess ventriculo-vascular characteristics using cardiac magnetic resonance (CMR) derived aortic area strain (AS) in conjunction with left atrial (LA) and left ventricular (LV) longitudinal and circumferential strain (LS and CS).

## Methods

Seventy five subjects, including 50 patients with repaired CoA divided into hypertensive (n=25), and normotensive groups (n=25), and 25 healthy subjects (controls) were studied. Hypertension was defined per National High Blood Pressure Education Program Working Group guidelines as systolic blood pressure (SBP) ≥95th percentile. For aorta acquisitions, through-plane, free-breathing, phase-contrast CMR protocol was used and AS measured at 3 levels: ascending aorta, proximal descending aorta and descending aorta at diaphragm. Maximal and minimal luminal areas (mm^2^) were measured, and AS (%) was calculated as 100 X (Area max - Area min)/ Area min). LA and LV LS were measured from horizontal long axis steady state free precession cine (4-chamber image) and LVCS from mid-ventricular short axis cine using CMR-feature tracking (6-segment model, 2D-CPA, TomTec). LA and LV end-diastolic volumes (EDV), ejection fraction (EF) and LV mass were also measured (Medis) in all subjects. Measurements were compared between the 3 groups.

## Results

**Figure 1:**
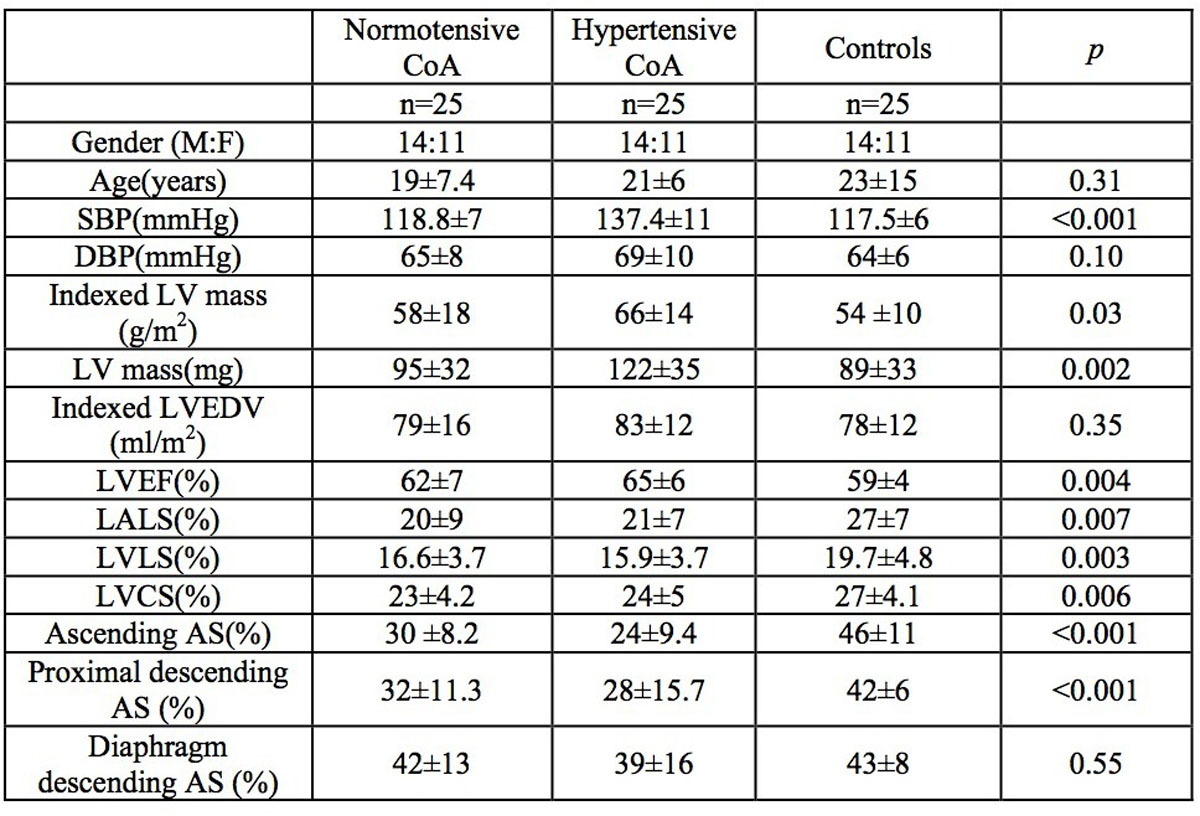
Figure 1

The mean age of CoA patients were 19.7 ±6.7 years (range 8.5-43 years) and controls were 23 ±15 years (range 7.1-51 years). All strains (LA, LV, ascending and descending aortic) were significantly lower in CoA subgroups compared to controls (Figure 1) except the AS at diaphragm which was not different between CoA subgroups and controls. Comparisons between the hypertensive and normotensive CoA subgroups showed no statistically significant differences in indexed LV mass, LA and LV volumetric and strain indices, however the ascending AS was significantly lower in the hypertensive subgroup (p=0.02). Ascending AS was correlated with LV mass (r= -0.4, p=0.005), LVEF(r= -0.4, p=0.004), SBP(r= -0.5, p=0.000) and LVLS(r=0.5, p=0.001).

## Conclusions

Ascending AS in CoA correlated with LV mass, EF and LVLS. In hypertensive CoA subjects, ascending AS was significantly reduced compared to normotensive CoA and healthy controls of similar age and body surface area. These observations may be indicative of differences in vascular remodeling influenced by ongoing hypertension.

## Funding

Childrens Hospital and Medical Center Foundation, Omaha, USA.

